# Microgel that swims to the beat of light

**DOI:** 10.1140/epje/s10189-021-00084-z

**Published:** 2021-06-15

**Authors:** Ahmed Mourran, Oliver Jung, Rostislav Vinokur, Martin Möller

**Affiliations:** 1 DWI - Leibniz-Institut for Interactive Materials, RWTH university, Forckenbeckstr. 50, D-52056 Aachen, Germany; 2 Institut of Technical and Macromolecular Chemistry der RWTH Aachen, Forckenbeckstr. 50, D-52056 Aachen, Germany; 3 3 Max-Planck School Matter to life, D-69120 Heidelbergy, Germany

## Abstract

**Abstract:**

Complementary to the quickly advancing understanding of the swimming of microorganisms, we demonstrate rather simple design principles for systems that can mimic swimming by body shape deformation. For this purpose, we developed a microswimmer that could be actuated and controlled by fast temperature changes through pulsed infrared light irradiation. The construction of the microswimmer has the following features: (i) it is a bilayer ribbon with a length of 80 or 120 $\upmu$m, consisting of a thermo-responsive hydrogel of poly-N-isopropylamide coated with a 2-nm layer of gold and equipped with homogeneously dispersed gold nanorods; (ii) the width of the ribbon is linearly tapered with a wider end of 5 $\upmu$m and a tip of 0.5 $\upmu$m; (iii) a thickness of only 1 and 2 $\upmu$m that ensures a maximum variation of the cross section of the ribbon along its length from square to rectangular. These wedge-shaped ribbons form conical helices when the hydrogel is swollen in cold water and extend to a filament-like object when the temperature is raised above the volume phase transition of the hydrogel at $32\,^{\circ } \hbox {C}$. The two ends of these ribbons undergo different but coupled modes of motion upon fast temperature cycling through plasmonic heating of the gel-objects from inside. Proper choice of the IR-light pulse sequence caused the ribbons to move at a rate of 6 body length/s (500 $\upmu$m/s) with the wider end ahead. Within the confinement of rectangular container of 30 $\upmu$m height and 300 $\upmu$m width, the different modes can be actuated in a way that the movement is directed by the energy input between spinning on the spot and fast forward locomotion.

**Graphic abstract:**

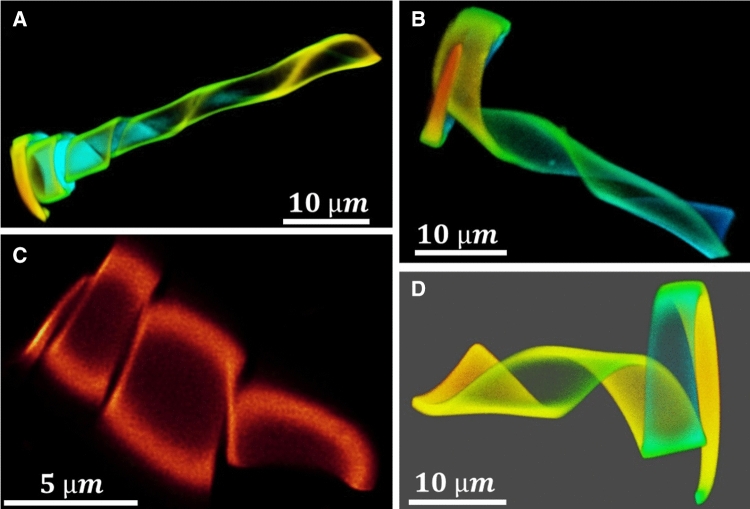

**Supplementary Information:**

The online version contains supplementary material available at 10.1140/epje/s10189-021-00084-z.

## Introduction

Self-propelling microorganisms [1] such as Euglena, a single-cell flagellate eukaryote, move by large amplitude periodic shape deformations. Here, the term “large” implies an amplitude of a size of the whole organism. Interestingly, Euglenids can employ two distinct modes of locomotion. Swimming in a non-confined environment is effected by the beat of the flagellum with a regular beating period and a helical trajectory [2]. In a confined environment, the cell body undergoes a peristaltic motion, also called metaboly, which again is periodic and highly coordinated [3]. In both cases, locomotion involves a deformation of the body or its appendages in an environment where viscous forces are dominant, characterized by a low Reynolds number [4, 5]. Microorganisms cope with this constraint by deformation of their body in a hysteresis sequence [6], which is in fact one of the challenges in the design of periodically actuated microscopic swimmers. Reconstruction of the flagellar beat as well as the metaboly motion requires a detailed spatiotemporal resolution of the complex deformations and has been subject of physical modeling [3, 7]. Yet, it remains a challenge to image these shape deformations in detail with current microscopy techniques and recent reports focus on the evaluation of the motion trajectories [8–12] as a basis for the reconstruction of the propelling shape deformation. In contrast to these advancements, mimicry of such locomotion by synthetic objects that allow for reduced complexity and improved control of such body shape deformations is still in its infancy. It might contribute significantly to the understanding of the efficiency of such locomotion [13, 14], but will also provide new capabilities for microrobotic systems that can search their own way to transport and deliver, e.g., by chemotaxis as the motility is effected by gradient in concentration, or which can mix, sort and circulate fluids [1, 15].

Recently, we have described preparation and motility of helical ribbons that were actuated by periodic pulse irradiation with near IR-light [16–19]. We note that ribbons are long narrow strips possessing three distinct material length scales (thickness, width, and length) which produce unique shapes unobtainable by wires or filaments. Our ribbons were typically a few tens of micrometers long, one micrometer thick and had a width of 5 micrometers. They were prepared from a thermo-responsive poly(*N*-isopropylacrylamide) (PNIPAm) hydrogel whose solubility decreased with temperature [20], causing volumetric shrinkage. Another design feature of these ribbons is that they were heated from inside by means of near infrared light-absorbing gold nanorods (AuNRs) embedded in the hydrogel matrix. As a consequence, the surrounding medium acted as a heat sink. These slender bodies fulfilled specific requirements for swimming by large periodic shape deformations, such as (i) cyclic beating motion because the swelling-collapse deformation was caused by the periodic irradiation, (ii) peristaltic motion, as the light-induced volume collapse and bulge (wrinkles) were spatiotemporal inhomogeneous, and (iii) large deformation as inhomogeneous volume changes resulted in large amplitude bending motions. We could also demonstrate a strong asymmetry of the sequence of shapes during one cycle or beat. This remarkable behavior was assigned to the fact that heating and cooling of the microgel ribbons occurred faster than the corresponding volume change. Therefore, volume-temperature equilibrium state is not reached, and the deformation became path and shape-dependent.

We capitalize on our previous work to develop swimming microgels capable of navigation. This entails a proper choice of the shape, i.e., the design of the microgel objects must distinguish a head and a tail. For navigation, we want to control the motional mode by the light intensity and the beat period and study how the motion can respond to an obstacle or confinement. Here, we describe how this can be achieved with wedge-shaped ribbons with ends of different width. Upon photothermal heating, the variation in width causes different dynamic responses in volume change and bending along the centerline of the ribbon, which controls the locomotion. In the first part of this report, we describe the temperature-mediated variations of the 3D shape of the microgel ribbons under equilibrium conditions, i.e., for varying temperatures and corresponding degrees of swelling. Because of the bilayer structure, the ribbons respond to swelling by bending and the taper in width induces distinct coiled shapes. This point gives a first insight into the interrelation of the geometry and the mechanics of our elongated ribbons. In the second part, we focus on the shape variation under non-equilibrium conditions, when the wedge-shaped ribbons are periodically actuated by NIR light which leads to conditions where the temperature change is faster than the diffusion-controlled swelling and contraction. Finally, we discuss how the motility can be controlled by the irradiation pulse frequency between moving forward and spinning on the spot. We also analyze the resulting flow in the surrounding water that causes a forward swimming motion.

## Results and discussion

### Shape variation by swelling and consecutive bending

**Fig. 1:**
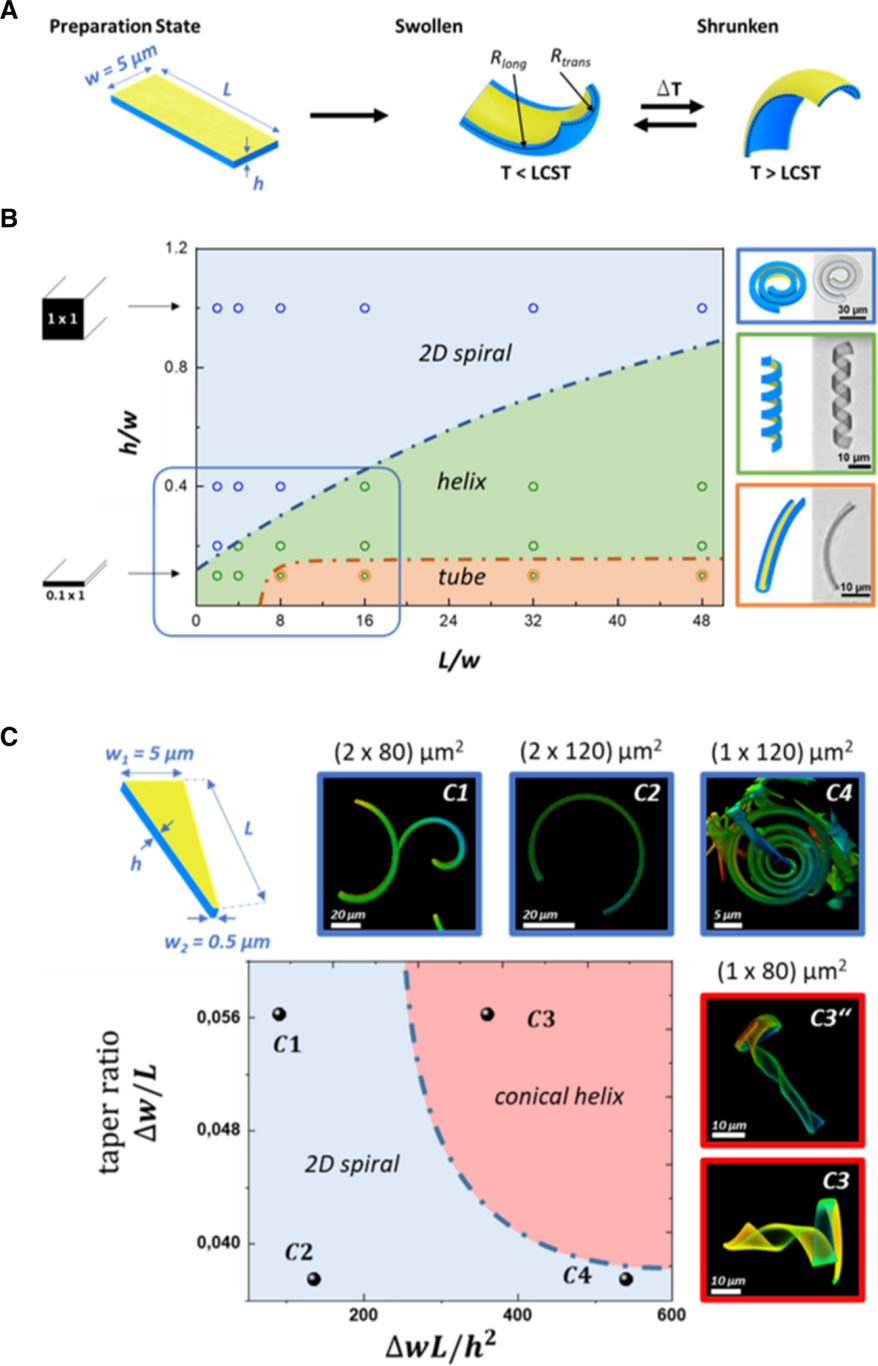
**A** The scheme shows a bilayer hydrogel ribbon with a thin gold layer (two nanometer-thick layer applied by sputter coating). At low temperature, this inextensible layer restricts the swelling of the PNIPAm hydrogel and causes the ribbon to bend biaxially inward. At high temperatures, the curvature is reversed. **B** Shape diagram of swelling caused conformations of rectangular bilayer strips of varying length *L* (x-axis) and thickness *h* (y-axis), normalized to a constant width of $w =\textit{ 5 }~\upmu \hbox {m}$. For a square cross section, the ratio of thickness to width is equal to unity ($h/w=1$). Points in the graph present the conformations, we found experimentally for the equilibrium-swollen structure at $20\,^{\circ }\hbox {C}$. Discontinuous lines are hypothetical boundaries between different structures. **C** Conformations of bilayer ribbons that were cut in a wedge shape along their length is illustrated by the drawing. Points in the graph denote experimentally found equilibrium structures in water at $20\,^{\circ }\hbox {C}$ shown in the corresponding confocal micrographs: $\hbox {C}1 (2\times 80)~\upmu \hbox {m}$, $\hbox {C2} (2\times 120)~\upmu \hbox {m}$, $\hbox {C4} (1\times 120)~\upmu \hbox {m}$, and C3, C3” are $(1\times 80)~\upmu \hbox {m}$ (additional structures are shown in Fig. 2). We limited our investigation to two lengths of 80 and $120~\upmu \hbox {m}$, respectively, as well as two thicknesses of $1~\upmu \hbox {m}$ and $2~\upmu \hbox {m}$. The width of the wedge-like ribbons was altered from $5~\upmu \hbox {m}$ at the wider end to $0.5~\upmu$ m at the narrow end. The graph plots the taper ratio of the ribbon against Föppl–von Kármán number as a geometric parameter accounting for the tendency of thin sheets to bend. The dashed line is a hypothetical boundary between spiral and conical helix

A key element of our experiments is given by the transformation of volume changes to large amplitude bending. In order to achieve this, we made thin hydrogel ribbons with a bilayer composition. As described before, the ribbons were prepared by micro-molding and UV-crosslinking of *N*-isopropylacrylamide and *N,N*’-methylene-bis(acrylamide) dissolved in DMSO [21]. For the design of the geometry, we used lithographic techniques. An elastomer made from perfluoro-polyether was employed for the fabrication of the mold [22]. At $32\,^{\circ } \hbox {C}$, the hydrogels undergo a volume phase transition due to lower critical solution behavior. In order to impart a temperature-dependent bending, the polymer ribbons were sputter coated on one side by a 2-nm-thick layer of gold when they were still in the mold. When such bilayer is released into cold water, the hydrogel objects swell and expand relative to the metallic layer. Obviously, stress accumulation at the metal/hydrogel interface causes bending toward the side of the gold layer as shown in Fig. 1a. Remarkably, an inversion of the curvature occurs when the hydrogel transforms from the swollen state at low temperature to the collapsed state at elevated temperature.

Modeling of bilayer bending as a consequence of equibiaxial misfit strains is well-established in the literature [23–26]. As long as the surface of the bilayer sheet is kept constant, bending in two directions must be isometric, i.e., the Gaussian curvature $\kappa$ is constant. This condition is fulfilled if one of the principle curvature is zero, $\kappa = 0$. As consequence, the expansion or shrinkage of a rectangular hydrogel/gold-film bilayer tends either to form a long tube (bending around the long axis of the rectangular ribbon) or a spiral (bending normal to the width of the ribbon). Elasticity of the bilayer structure enables biaxial bending, $\kappa > 0$, corresponding to some bulging as shown in Fig. 1a. For large curvatures, however, the energy required for elastic deformation is too high and the ribbon only bends around one direction (tube or spiral). Limited biaxial bending can still be found at sides of the ribbon. Because these double bending sides are longer in the case of a spiral, formation of a spiral is energetically more favorable for a long ribbon. This edge effect causes the ribbon to wind up as a helix, with the helix direction controlled by small misfits of the geometrical long axis and the stresses which result from imperfections in geometry and homogeneity of the ribbon [25]. Figure 1b demonstrates such shape variations for our rectangular hydrogel/gold-film bilayer samples with different length-to-width-to-height ratio at equilibrium swelling at $20\,^{\circ } \hbox {C}$, i.e., at fixed swelling degree. For the shape diagram in Fig. 1b, we normalized the length and thickness to the width $w =5~\upmu$m. As expected, the ribbons form spirals which transform into helices when the aspect ratio was increased or when the thickness was reduced. Unexpectedly, we observed formation of tubes for thin and long ribbons although spiral conformations should be preferred at high aspect ratios. However, the evolution of a tube may be guided by irregularities in the swelling process, e.g., variations in height along the ribbon, release from the mold, or dog ear formation at the end of the ribbon. Spontaneous curvature perpendicular to the long axis will create a geometric obstruction or barrier to reaching the spiral shape. The tubes, which we observed, were bent like a banana demonstrating some biaxial bending influence. Important for this work here is the transition from a spiral to a helical conformation, whereas the main principle curvature is controlled by the h/w ratio. For a square cross section ($h/w=1$), the bending around the transverse direction dominates, while small h/w ratios favor the helix formation.

**Fig. 2:**
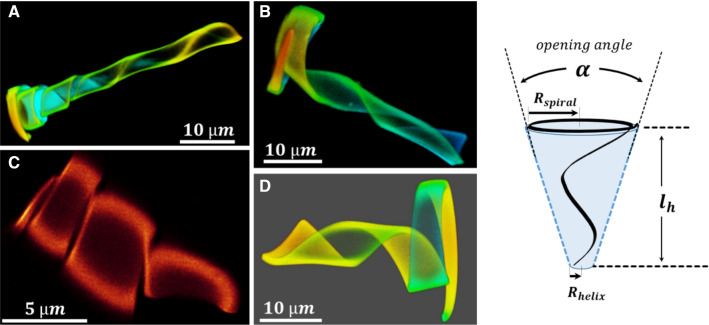
Four typical 3D cone conformations ($20\,^{\circ }\hbox {C}$ in water) which were obtained by one microlithographic fabrication batch. The batch was directed to yield ca. 50% ‘identical’ hydrogel ribbons of $80~\upmu \hbox {m}$ in length, $1~\upmu \hbox {m}$ height and a width of $5~\upmu \hbox {m}$ and $0.5~\upmu \hbox {m}$ at the two ends. However, as the images demonstrate, small variations in the dimensions of the ribbon can cause significant differences in the 3D conformations. We classified these variations qualitatively by four groups: Type A, a helix that widens at the end to a short spiral element; Type B and D: a widening helix with a spiral at the end whose length is roughly identical to the length of the segment that forms the helix; and Type C, only a helical element. The drawing shows the parameters that describe the conical helix. The images refer to other figures as follows: $A = C'''; B = C3''; C = C'; D = C3$

When the bilayer hydrogel ribbon is cut to a wedge shape, the bending preferences varies along the length of the ribbon and can span different areas of the diagram in Fig. 1b. The insets C3 and C3” in Fig. 1c depict confocal microscopy images, showing that such objects can combine spiral and helical elements, which results in the formation of a cone-like 3D conformation. As the cross section varies from square to rectangular along the ribbon long axis, the transverse bending becomes more important, favoring a 3D helical over the 2D spiral shape [27]. For the samples C1 and C2, the equilibrium shape is a two-dimensional spiral in which the narrow tip bends tightly, while the wider end opens radially. When the thickness was reduced, the curvature increases resulting in a spiral structure similar to a clockwork spring, as shown in image C4 of Fig. 1c. C3 in Fig. 1c shows the transition from a spiral to a helix. Hence, the images demonstrate a remarkable shape variation between the ribbons and even within one ribbon. Note that in the case of a 2D spiral, the narrow end was oriented to the core of the spiral where the curvature is maximal (C4). When, however, the ribbon adopted the 3D cone conformation, obviously controlled by the curvature of the wider end, its narrow end pointed to the outside of the spiral that developed at the base of the cone (C3, C3” including all the conical configurations in Fig. 2). In order to classify the different conformations, we plotted the taper ratio (*w*/*L*) of the ribbon against Föppl-von-Kármán number ($wL/h^{2}$) which is a geometric parameter determining how easily a sheet bends [28, 29]. The dashed line is a hypothetical borderline between the spiral objects and the 3D cone objects. In the case of the ribbon in C3 with a length of $80~\upmu \hbox {m}$ and a thickness of $1~\upmu \hbox {m}$, the narrower end only bends around the transverse axis (uniaxial bending), while the conformation at the wider end gets controlled by the longitudinal and transverse axis (biaxial bending). This approach to data presentation was derived from the consideration that the 2D spiral should be controlled by the gradient in curvature and, as the ribbon width is increased, bending in the transverse direction plays an increasing role and results in an apparent stiffening of the ribbon [30], which we consider to be proportional to the gradient in width $\Delta \hbox {w/L}$ [31]. So far, we limit our analysis of the bending of the wedge like ribbons to the description of the experimental observation. Rigorous modeling of the bending is beyond the scope of this work with its focus on the non-equilibrium actuation.

The examples demonstrate how the stresses caused by swelling can be exploited to realize distinct geometric variations of the shape of such hydrogel objects. Actually, we found that the conformation of the objects is susceptible to structural variations that are even less pronounced. The accuracy of the micro-molding has a variance of about 100 nm [32] (mainly determined by templating a silicon micromold to a perfluorpolyether mold, from which the object is obtained). Relative to the small thickness of the ribbons of 1 or $2~\upmu \hbox {m}$, this can cause significant variations in bending. Figure 2 shows four 3D cone conformations observed for four wedge-shaped ribbons that have been prepared in the same fabrication batch ($80~\upmu \hbox {m}$ long, $1~\upmu \hbox {m}$ thick and a width of 5 and $0.5~\upmu \hbox {m}$ at the two ends resp.) The comparison demonstrates that the combination of a helical and a spiral element is strongly influenced by small variations, which we cannot control so far. However, because we can study the temperature response of such objects individually, these variations enable us to observe their effects on the motility or to find efficient swimmers, as it will be shown below.

### Temperature response and NIR-light actuation

**Fig. 3:**
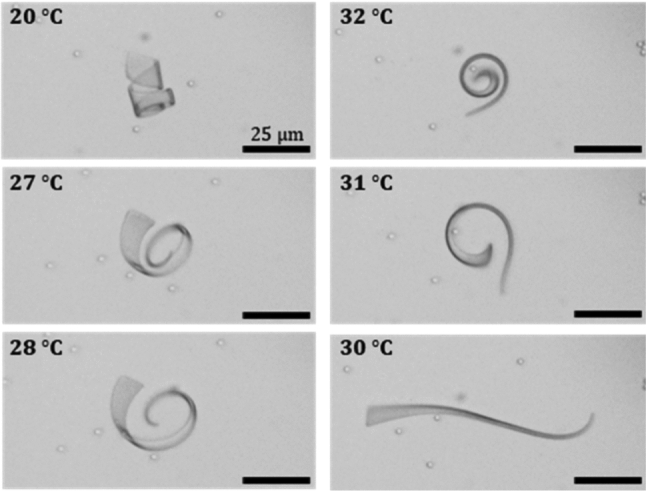
Optical micrographs of a tapered bilayer ribbon, $80~\upmu \hbox {m}$ long and $1~\upmu \hbox {m}$ thick, whose shape changes during a quasi-static rise in temperature. At $30\,^{\circ }\hbox {C}$, close to the volume phase transition temperature, the ribbon unwinds whereby the wider end begins to bend in the opposite direction than the tip. Above $30\,^{\circ }\hbox {C}$, the curvature gradually reverses

So far, we looked at the equilibrium state of the wedge-shaped ribbons in water at low temperature as caused by the swelling of the hydrogel. Figure 3 shows a series of optical microscopy images at raising temperature obtained for sample C in Fig. 2. As the degree of swelling decreased at elevated temperature, the two ends changed their shapes in different ways. The spiral end became straight, while the wider end even underwent the first steps for a helix inversion. Hence, the design of a wedge-shaped bilayer ribbon does not only demonstrate how different shape deformations can be combined within the body of single object making a distinction for its “head” and “tail”, but both ends of the wedge-shaped ribbon respond also differently to temperature changes. Together, these two features are helpful but not sufficient to cause locomotion of such small objects.

As mentioned in the introduction, directed motility of such micro-objects in water, i.e., swimming, occurs at low Reynolds numbers and requires a cyclic but asymmetric or hysteretic sequence of shapes. Such a hysteresis-shape response is only found under non-equilibrium conditions. Theoretical models for microswimmers that undergo locomotion by shape deformation are based on a retarded volume or shape response, e.g., by assuming instantaneous temperature changes [33] or by snap buckling [34]. In other words, out of equilibrium actuation ensures that the shape deformation cycle does not comprise time reversible steps. Consequently, a net flow in the surrounding fluid is generated, resulting in a displacement of the body. In the case of a thermo-responsive hydrogel, hysteresis of the heating and cooling response can be expected at fast heating and cooling rates, whereby stress generation is coupled to the diffusion of the water out of and into the gel objects. Thus, transient stresses are built up within the gel because its volume cannot respond quickly enough to the temperature [35–37].

In our work, we used gold nanorods as heating elements inside the microgel objects for the required fast raise in temperature. Typically, we incorporated nine nanorods per cubic micrometer of the gel. Their average dimensions are 60 nm length and 15.4 nm diameter [16]. These nanorods are superior heating elements when they are irradiated by NIR-light. They convert the incident light to heat nearly by 100% within nanoseconds [38, 39]. Thus, the heating rates are controlled solely by the thermal diffusion in the aqueous gel and thus mainly by the average distance between Au nanorods in the gel particles and the light intensity. By light pulses, the temperature can be raised by several degrees within milliseconds. When the IR-irradiation stops, the small microgel objects cool down rapidly as the heat is transferred by the same thermal diffusion to the surrounding water, acting as a huge heat sink [40].

Our previously reported rectangular helical microgel ribbons had a constant radius [17]. When heated by NIR-light pulses both ends reacted the same way. (The irradiation was almost homogeneous over the whole sample.) Heating resulted in unwinding and even reversal of the helix. The cyclic deformation showed a hysteresis in the shape sequence upon fast heating and cooling, as the actuation occurred at non-equilibrium conditions [16, 19]. When confined between two planes, the gel objects rotated around their long as well as around their normal axis. When one end touched a flat wall, we observed a net translation along this wall.

**Fig. 4:**
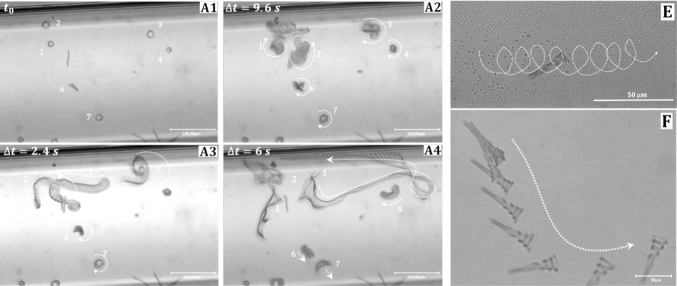
**A1–4**) A low-magnification optical micrograph reconstructed by the superposition of images at different time intervals enabling tracking the trajectories of different helical swimmers in this case were weakly confined as the gap was $30~\upmu \hbox {m}$ high, thus larger than the helices dimensions. The period was 11 ms whereby irradiation was during 3 ms and the recovery for 8 ms (see SI Video 1). The image shows three conical helices of the type D in Fig. 2, marked by numbers (1–3). When the conical helix got oriented with its wider part toward the glass wall, it spins at the spot (situation A1–2 in the image), whereas when it moves forward, the velocity was roughly $500~\upmu \hbox {m}/\hbox {s}$ (situation A3–4). **E** Shows the motion trajectory of a Type B conical helix, which resulted from the combined rotational and translation modes when irradiating for 1 ms and 8 ms recovery cycles. **f** The object resembles the one in Fig. 2b/c. Also this one moves forward with its wider part ahead. Different than the one in **A1–4**, it could not change its direction abruptly and just turned in a wide bend as a consequence of a forward motion and rotation, most likely because of the effect of the two glass walls. Obviously **e**, **f** demonstrate rather stable modes of motion within the confined space of the rectangular capillary. SI Video 2 and 3)

Because the new conical hydrogel objects reported here have distinguished ends, we expected to observe a forward motility even under non-constrained conditions, when their shape variation was actuated by fast heating–cooling cycle under non-equilibrium conditions. This is indeed the case as shown in Fig. 4 and the corresponding movies in the supporting information (SI Movie 1–3). Figure 4 depicts three examples of ribbons that formed conical helices in water at $20\,^{\circ } \hbox {C}$. They were observed swimming in a rectangular capillary with a $300~\upmu \hbox {m}$ wide and $30~\upmu \hbox {m}$ high lumen when they were actuated by short NIR-light pulses separated by a defined recovery time. Taking into account that the length of the conical helices is between 10 and $50~\upmu \hbox {m}$ and that the extended ribbon spans $80~\upmu \hbox {m}$, the hydrogels are confined in their movement parallel to the direction of observation. They move rather freely within the observation plane, but they cannot freely turn around the normal axis. Within the gap between the upper and the lower glass wall, we observed, however, that they can swim against gravity and their mode of motion could change, when they encounter the upper wall. In some cases, we observed hydrogel objects spinning at the spot as they hit the glass-wall (Fig. 4 A1). In other cases, the conical objects swam forward within the horizontal plane. Figure 4A 3–4 demonstrates the forward motion of cones with a low-temperature conformation like the one shown in Fig. 2D with the spiral end ahead. When the hydrogel got oriented with its cone axis perpendicular to the top/bottom side of the capillary it got stuck for some time (SI movie 1) as the wider part is moved against the wall (Fig 4A 1, 2, 3). Eventually, the hydrogel could escape this situation and started to move forward parallel to the horizontal capillary walls with a displacement velocity of ca. $500~\upmu \hbox {m}/\hbox {s}$ (6 body lengths per second). A different response is shown in Fig.  4e and SI movie 2 for A/B type conical helix (see Fig. 2). Clearly, the object moved also with its wider part ahead but it followed a cycloidal trajectory. A different situation is shown in Fig. 4f for an object that resembled the one in Fig. 2a/d. Also this one moves forward with its wider part ahead. Different than the one in Fig. 4a, it could not change its direction abruptly. However, it follows a curved trajectory as the consequence of a forward motion combined with rotation. Obviously Fig. 4e, f shows rather stable modes of motion within the confined space of the rectangular capillary.

**Fig. 5:**
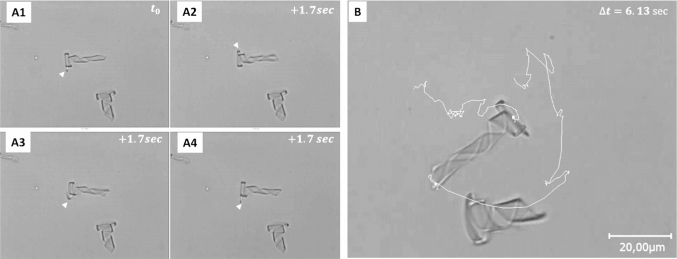
**A1–4** sequence of images of the conical helix shown in Fig. 2d, when actuated by 200 $\upmu$s long NIR-pulses followed by a recovery time of 800 $\upmu$s. The time between images was 1.7 s, which corresponds to 5170 light pulses during which the helix spins counter-clockwise around the helical axis and makes one revolution. **b** Trajectory of the helix after 67 s indicating that while it spins, it translates and rotates clockwise. Note that the motion is strongly biased by the glass surface because, in this case, the capillary was only 20 $\upmu$m high. (The corresponding video is presented in the Supporting Information, movie SI 4.)

The examples demonstrate for all three conical helix objects a common forward motion component with the wider end as the head. They also show that small variations in the geometry in combination with the spatial confinement can cause sideward and rotational components of the motion. Figure 5 shows a series of stroboscopic images of the light actuated object displayed in Fig. 2D. They were observed swimming in a rectangular capillary with a $200~\upmu \hbox {m}$ wide and $20~\upmu \hbox {m}$ high lumen. In this case, the cone could not orient perpendicular to the wall. This results in sudden reorientation of the micro-object when it collides with the walls. We did not observe any adhesive contacts, which would permanently tether the helix to the glass. Clearly, the helix turns in clockwise direction, most likely because of the leverage effect of the friction at the wider spiral head. Such a directed rotation can easily explain the forward motion in analogy to the rotating flagella of bacteria such as Escherichia coli [4].

**Fig. 6:**
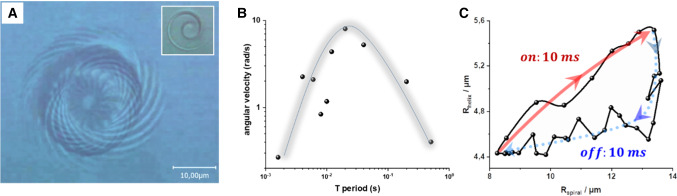
**a** Overlay of optical micrographs that demonstrates the clockwise rotation of a conical helix with its long axis oriented perpendicular to the capillary wall above it when it has been actuated by light on and light off sequences of 10 + 10 ms. This orientation is kinematically preferred when the actuation is chosen to maximize the amplitude of the oscillation whereby the rotation velocity is maximum. The axial length of the conical helix was $30~\upmu$m, and the glass capillary was $30~\upmu$m high and $300~\upmu$m wide. The main image shows a superposition of the conical helix as it rotates clockwise. The outer radius marks the trajectory of the head of the conical helix, while the inner radius marks the trajectory of the tail. The inset contains a snapshot of the view along the axis of the conical helix through the wide end which is close to the wall. **b** The variation of the angular velocity depending on the irradiation period. **c** The radius of the tail $\hbox {R}_{\mathrm{helix}}$ (or inner radius) as a function of the radius of the wider head

### Control of the motility by the energy input such as the NIR-irradiation sequence

So far, we have demonstrated the differentiation between a head and a tail of the conical helix and shown that it can be actuated to undergo directed motion by pulsed NIR-light. Going a step further, we hypothesize that the coupling of different motional modes within one object can be controlled by the pulse rate and the intensity of the NIR-irradiation. As a consequence, we expected that the motion can be directed by the choice of the pulse sequences. In Fig. 4, we depicted the motility at relatively short irradiation pulses (3 ms on/8 ms off). Within the confinement of a flat channel, we observed a fast lateral swimming mode, during which the helix got practically fully extended (**Sequence** Fig. 4A3–4). Figure 6 and the corresponding movie (Supporting Information SI-movie 5) demonstrates the movement of such a conical helix under irradiation (10 ms on/10 ms off). The helix axial length was $30~\upmu \hbox {m}$ with a radius of the spiral at the wider end of $12~\upmu \hbox {m}$ and $4~\upmu \hbox {m}$ for the radius of the helix at the narrow end. It is shown in a situation when it got confined in one direction as the conical helix oriented perpendicular to the upper wall of the capillary. As already shown in Fig. 4 and the corresponding video, this blockade of a forward motion restricts the cone to rotate slowly around its long axis while the spiral end and the helix unwind and wind during the heating–cooling cycles. The vertical orientation enabled us to track the rotation of the extremities of the cone around its long axis and thus to measure the time dependence of the cone’s radii. The inset in Fig. 6a depicts the view along the axis of the truncated coil through the spiral end which is in contact with the wall. The main image in Fig. 6a shows a superposition of the cone as it rotates clockwise without changing its position during an actuation cycle of 20 s. The outer radius marks the trajectory of the end of the spiral, while the inner radius marks the trajectory of the eye of the helix. Variation of the sequence of actuating light pulses effects the amplitude of the opening of the coil. When we chose the irradiation frequency in a range so that the cone remained entrapped in its orientation, we could analyze the rate of the rotation around the long axis of the cone. The variation of the angular velocity for different irradiation sequences is shown in Fig. 6b (light-on and light-off times were kept identical). The double-log diagram indicates that the rate of the motion can be regulated over a range of two orders of magnitude from roughly one revolution per minute at low and high frequencies to more than one revolution per second for the 20 ms actuation cycle. Figure 6c shows how the radii of the head and the tail varies during a full actuation cycle for the 20 ms actuation period, which corresponds to the maximum amplitude of the opening of the helix. The diagram demonstrates a more or less simultaneous opening of the helix and the spiral during heating, while the helix segment re-twisted almost instantaneously when the light was switched off accompanied by comparable slow rewinding of the spiral. Clearly, the deformation cycle is non-reversible with the area under the curve being related to the work produced by the rotor.

**Fig. 7:**
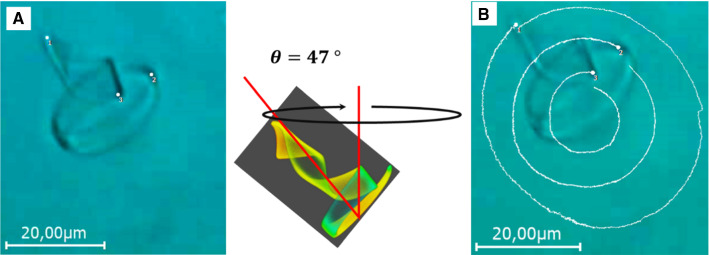
**a** Inclined configuration of the conical helix with respect to the glass surface. **b** Shows clock-wise rotation path after 24 s under irradiation with $800~\upmu$s pulse and $800~\upmu$s recovery time. The rotation velocity was 23 $\hbox {deg}/\hbox {s}$. The drawing illustrates the tilt angle

Figure 7a, b and the SI movie 6 show the movement of the same conical helix, while it was tilted toward the capillary wall by an angle of $47\,^{\circ }$ [41]. In this sequence, the light actuation period was 1.6 ms. Under these conditions, the amplitude is so small that the helix conformation appears optically unchanged. However, it moved steadily around an imaginary axis with the head oriented inward. Figure 7a shows the tracking points (white dots) to trace the rotation of the helix. The white traces depicted in the overlay image 7B show the trajectory of these points after a full rotation around the imaginary axis, which lasted about 24 s, taking 15,000 actuation cycles. Due to the different displacements during one actuation cycle of the ribbon at the head and the tail, and the corresponding friction effects on the fluid, a torque is created (on the fluid) causing a clockwise rotation of the helix around its axis.

**Fig. 8:**
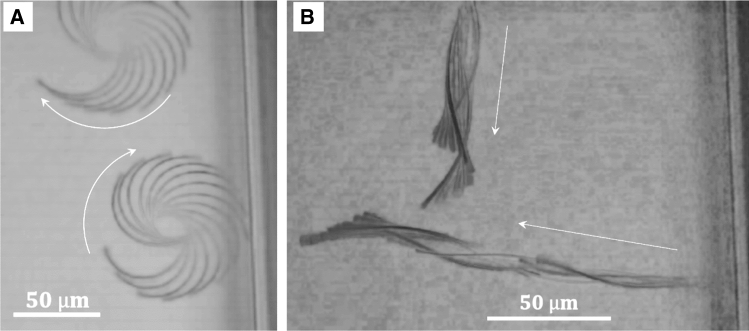
**a** Overlay of images with an interval of 300 ms showing the typical deformation and the trajectories of the tapered ribbon, while it spins clockwise under an irradiation period of 10 ms (2 ms on and 8 ms recovery), **b** while for a strobe period of 12 ms (4 ms irradiation and 8 ms recovery), it translate by undulation of the body, in this case overlay images with an interval of 396 ms it helps to show a wavelike motion. (See the corresponding movie 7)

As mentioned above, the confined situation displayed in Figure 6a is characteristic for the 20 ms irradiation period at the maximum of the deformation amplitude and rate. At short irradiation times, the small deformation amplitude did not stabilise the spinning of the helix with its axis orthogonal to the surfaces. The orthogonal orientation is also less stable for longer irradiation periods. Actually, the 20 ms period coincides with the timescale of the diffusion of the water molecules in and out of the gel [42]. Apparently, the orthogonal orientation of the helix is stabilized only because of the actuation (kinematic stability) and that it should be possible to direct the locomotion by changing the pulse period. That is indeed possible, as demonstrated in the following. Figure 8 presents an example, how the swimming can be directed by the modulation of the irradiation. In Fig. 8a, the time-dependent superposition of the images of two conical helices is depicted as they were irradiated by a 2 ms *on* and 8 ms *off* sequence. (The time between two images was 300 ms.) At this irradiation, the conical helix opened only slightly and rotated around its long axis, alike those shown in Fig. 6, whereas the time for a full rotation lasted 18 s. The image sequence displays different positions of the arc of the spiral as it rotates in one case clockwise and in the other case counter-clockwise. Whether the conical helix rotates clockwise or counter-clockwise is controlled by the handedness of the helix which in turn is determined by small asymmetries in the structure of the wedge shaped ribbon [16]. When the irradiation time was increased from 2 to 4 ms, while the cooling time was kept constant at 8 ms, the ribbon unwound to nearly full extension and did not have sufficient time to recover its helical conformation. Instead it started to wag like a flagellum, obviously combined with a rotation around its long axis. The main image in Fig. 8b presents the observation of the forward movement of two of such ribbons actuated in their extended state. We displayed a superposition at different times with a time interval of 396 ms. Clearly, the movie demonstrates the forward swimming of the extended ribbon with its wider end ahead at a velocity of $20~\upmu$m/s. The example shows how the rather unpretentious geometry of the wedge like bilayer ribbon in combination with a light actuated shape transformation induce directed motility of these micro-objects and how this motion can be stirred in a remarkable way by the control of the energy input. By the choice of the irradiation sequence, we could command the agitated ribbons to rotate around at a place and order them to swim forward when the irradiation sequence was switched. Here, the velocity of the forward motion resulted to $20~\upmu$m/s (1/2 body length/s), which is comparable to the non-confined swimming of a Euglena [2].

**Fig. 9:**
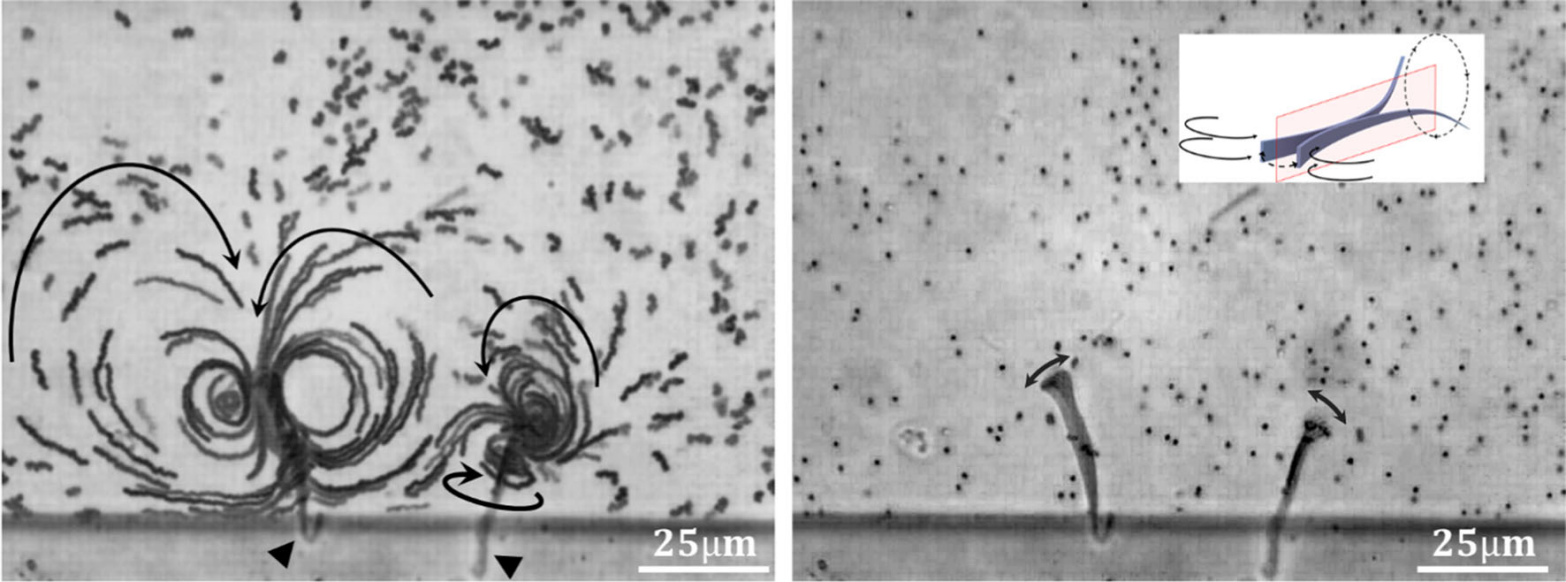
Flow direction and the streamlines pattern of tracer microparticles generated by two tethered tapered ribbons during actuation (4 ms pulse and 12 ms recovery). The triangles indicate where the ends got bound to the polydimethylsiloxane wall of the compartment. Left: the ribbon was attached by its narrow end and the arrows describe the direction of the flow, Right: the ribbon was attached by its wider end and the arrows describe the direction of movement. The inset illustrate possible streamline during actuation of a free ribbon (See the corresponding movie 8)

We have mentioned above that swimming of such thermoresponsive hydrogel micro-objects in Newtonian fluid requires a hysteresis motion cycle, which in turn entails shape deformation at non-equilibrium conditions. Such body deformation performs work on its environment because it causes a circulation of the surrounding fluid. We visualized the flow of the surrounding liquid for the swimmer in its forward moving state shown in Fig. 8b by following the traces of small colloidal particles. This has been possible as we have been able to observe the extended swimmer in action, while it was tethered either with its narrow end or with its wide end to a wall and could not move out of the image area.

In this case, we did not use a rectangular capillary for the microscopic observation, but watched the ribbons in a compartment imprinted in a thin layer of polydimethylsiloxane rubber that was sealed by a thin cover glass yielding a square water cavity of ($500 \times 500$) $\upmu$m$^{2}$ and 30 $\upmu$m in height. We assume that the ends were tethered to the edge of the silicone cavity by hydrophobic interaction and with the help of the cover glass.

Figure 9 shows two distinct patterns of flow lines induced by the periodic deformation of either the widest part (*left*) or the narrow tip (*right*) of the tapered ribbons which got tethered to the silicone edge by their opposite ends. For the free ends, we now can see the flow in the surrounding water. The arrows indicate the respective flow direction. The flow lines are clearly distinct and different for the two ends. Hence, they indicate different modes of motion for the wider and the narrower end of the wedge-shaped ribbon. While the wide end of the ribbon undergoes sideways flapping indicated by the very well-developed vertical vortices, the narrow end evidently demonstrates also a rotational component around the main axis of the ribbon combined with the wagging of the tail. In a first attempt, we describe the motion like it is depicted in the scheme in Fig. 9b. How and whether the coupling of theses modes of motion, flapping and rotational wagging could cause the forward motion in the direction of the wider end is a question beyond the scope of this publication. Certainly, the model in Fig. 9b gives a base for advanced mathematical modeling of the observed motility of the freely swimming ribbon.

## Conclusion

Complementary to the quickly advancing understanding of the swimming of microorganisms, we demonstrated rather simple design principles for systems that can mimic swimming by body shape deformation excluding propelling by a rotary motor. Firstly, and actually compelling by the laws of thermodynamics, the actuation must follow a non-equilibrium path. Secondly, directional swimming that distinguishes between forward and backward movement does not only require a swimmer with a ‘head’ and a ‘tail’ but should also involve coupling of different modes of motion. Thirdly, coupled modes of motion allow to control and direct the locomotion by the energy input modulation as the different modes can be actuated to different extent. All three points became possible by the fact that we could drive the motion from inside the object by plasmonic heating to which the gels respond by volume changes that transform to different bending modes. While these bending mechanisms are predefined by the bilayer structure of the ribbons, they are controlled by the deviation of the actuation from the equilibrium swelling/shrinking path.

Based on these points, we have been able to design a microswimmer that swims forward in the direction of the flapping end. This might appear counterintuitive; however, the forward thrust can only be understood by the coupling of this motion with the one of the tail. It may be speculated that the flapping head stabilizes the direction of the forward thrust of the tail. A more detailed analysis should be a subject of hydrodynamic modeling. The forward speed was determined up to 6 body length/s, which is comparable to the rates measured for bacteria *Thiovulum majus* [43].

It should be noted that the locomotion of our microswimmer is in fact not fully autonomous, but still controlled from the outside, i.e., by the energy uptake and thus the width of the irradiation pulses and the delay between them. A fully autonomous swimmer would require a mechanism such that the swimmer itself will control the switching between energy uptake and relaxation. This task has not been addressed here. For a light-driven swimmer this might be achieved by orientation or temperature-sensitive light absorption. Alternatively, the swimmers could be driven by an intermittent chemical reaction. Such a metabolic energy source remains subject of topical investigations [44, 45]. Most chemical transformations are too slow to cause non-equilibrium situations within small open objects when stress generation is diffusion limited [46]. Hence, chemical non-equilibrium in living organisms is typically controlled by membranes and pores [47]. An exception is given by coupling the oscillatory Belousov–Zhabotinsky (BZ) reaction to the volume change of gels and stress generation, whereas the reaction itself is heterogeneous in space [48], Within a gel beam, the oscillation can cause bending deformation [49], transport, and peristaltic pumping [50]. This behavior was modelled with multiphysics simulations of three-dimensional poroelastic systems coupled to the Oregonator model [51–53]. It has been shown that the propagation of a Belousov–Zhabotinsky reaction–diffusion front in a submillimeter gel disk induces local changes in the curvature of the disk that result in periodic 3D flapping [44]. It will be interesting how such autonomous and periodic buckling deformation propagating along the thin body could be combined with the multi-mode design of the swimmers described in this report [54].

## Supplementary Information

Below is the link to the electronic supplementary material.

Supplementary material 1 (pdf 872 KB)

Supplementary material 2 (mp4 3539 KB)

Supplementary material 3 (mp4 28555 KB)

Supplementary material 4 (mp4 3646 KB)

Supplementary material 5 (mp4 118816 KB)

Supplementary material 6 (mp4 170647 KB)

Supplementary material 7 (mp4 43205 KB)

Supplementary material 8 (mp4 78324 KB)

Supplementary material 9 (mp4 154294 KB)
