# Supplementary material for: Microgel that swims to the beat of light
Source: Eur Phys J E Soft Matter. 2021 Jun 15;44(6):79. doi: 10.1140/epje/s10189-021-00084-z (PMC8206062; doi:10.1140/epje/s10189-021-00084-z)
Supplement: Supplementary file 1 — Supplementary material 1 (pdf 872 KB) [file 10189_2021_84_MOESM1_ESM.pdf]

Experimental section

Microgel particles were fabricated by the so-called particle replication in a non-wetting template technique in short PRINT.[22] It is based on a template made of perfluoropolyether elastomer.

Preparation of the perfluoropolyether template: A cleaned microscope slide was placed on top of a diced silicon master (5 × 2.5) cm, produced by photolithography (AMO GmbH), where two pieces of two-layered parafilm (Bemis, total thickness of ca. 200 µm) were used as spacers. The Perfluoropolyether-urethane dimethacrylate liquid (PFPE, Mw = 2000, Fluorolink MD700, Solvay Solexis) containing 1 wt% 2-Hydroxy-2-methylpropiophenone (Sigma Aldrich) was then injected into the space, ensuring its filling. Subsequently, it was exposed to a UV radiation (366 & 254 nm, 4 W at each wavelength, Konrad Benda) for 20 min under argon atmosphere. After curing, the PFPE elastomer was carefully peeled from the silicon master. The micro structured PFPE replicas were cut into suitable size of (6 × 6) mm^2. A flat featureless PFPE film with a thickness of 400 µm was prepared according to the same protocol.

Fabrication tapered bilayer ribbons laden with AuNRs: Crosslinker (N,N'-Methylenebisacrylamide, BIS, 99 %) and photo-initiator (2-Hydroxy-4′-(2-hydroxyethoxy)-2-methylpropiophenone, 98 %) were added at a molar amount of 1 % relative to N-isopropylacrylamide monomer which was recrystallized twice in n-hexane before utilization. A typical monomer solution thus contains NIPAM (575 mg), BIS (7.8 mg), photo-initiator (11.4 mg), and AuNR dispersion in DMSO (981 µL). The nanorods concentration was assessed by the optical density of the dispersion and it was 240 measured at the maximum wavelength of absorbance and an optical path of 1 cm. The typical number density was 9 nanorods/μm^3, with nanorods having a longitudinal absorption maximum at around 800 nm, very close to the laser irradiation wavelength of 808 nm. For fluorescent labeling, Nile red was added at a molar ratio of approximately 1:1000, relative to NIPAm. The fabrication procedure of microgel particles is the same as used in our previous reports.[21] A homemade press with a quartz window was used for the molding of microgel, and all steps were carried out in a glove box (with an oxygen content O_2 < 1 %). A drop of the monomer solution (0.5 µL) was deposited on the PFPE replica and subsequently covered by the flat PFPE-film. A weight was applied to generate suitable pressure (ca. 260 kPa) and ensure drainage of excess of monomer solution. This leads to filling of the microstructure and results in disconnected elements without residual film in between. Subsequently, the assembly was exposed to UV radiation (366 & 254 nm, 4 W at each wavelength, Konrad Benda) for 20 min. Subsequently, the weight was removed and the mold was peeled off from the flat film. The bilayer microgels were produced by sputtering a thin gold film (ca. 2 nm) while the microgels are still in the mold using a sputter coater (30 mA, 10 s, Edwards S150B). To transfer the samples, a droplet of 714 g/L

solution of Polyvinylpyrrolidone (PVP, 40 kDa) in ethanol/water (volume ratio: 7/63) was placed on a clean (25 x 25) mm cover glass, followed by the mold facing upside-down. The sample was then left to dry overnight.

Microscopic investigations and light actuation

For microscopic investigations, the PFPE-film was peeled and a droplet of ultrapure water (0.055 µS/cm, Purelab Plus UV) was applied to release the objects from the glass surface. The samples were then collected into plasma-cleaned (O_2, 40 mL/min, 200 W, 5 min, PVA TePla100) capillaries (Vitrocom) and sealed at the ends using two-component epoxy glue (high strength epoxy resin, RS Pro). The temperature was controlled with an accuracy of ±0.1°C, using a water cooled Peltier element mounted onto the microscope stage. Infrared LASER-irradiation (808 nm, 10 W, Roithner Lasertechnik) was applied at an incident angle of 45°, giving an elliptical spot size of approximately (2 x 1.5) mm. Videos with high temporal resolution of up to 1 ms were recorded using a digital microscope (VW-9000D, Keyence) with a high speed camera. The motion of the elements was tracked, using MotionAnalyzer (Keyence).

Confinement space and map of the flow field

An (8 x 8) array of square cavities, each with a width of 500 µm and a height of 30 µm was imprinted in PDMS rubber. To that purpose, a master was designed using computer-aided design (CAD) software (Inventor, Autodesk) and produced in a multiphoton lithography setup (Photonic Professional GT, Nanoscribe GmbH) with a 25x immersion objective (numerical aperture (NA), 0.8; Zeiss) and IP-S photoresin (Nanoscribe GmbH). The slicing and hatching distance were 1 µm and 0.5 µm, respectively. The laser power was set to 100 % and scanning speed at 100 mm/s. Substrate adhesion was enhanced by pretreatment of the glass slide with plasma (O_2, 40 mL/min, 200 W, 5 min, PVA TePla 100) and subsequent incubation overnight in a 1 % solution of 3-(Trimethoxysilyl)propyl acrylate (Sigma-Aldrich) in acetone. The structure was replicated in 0.2 mL PDMS (Sylgard 184, Dow Corning) using (300x300) µm square capillaries (Vitrocom) as spacers between the master and a glass slide to give a total film thickness of 600 µm. After removing the PFPE film from the sample, 4 µL of ultra pure water were pipetted to transfer it onto a plasma treated (20 x 20) mm Silicon wafer and left to dry. Afterwards, 1.5 µL of the microsphere tracer dispersion (FluoSpheres®, 1.0 µm, Invitrogen, diluted with ultrapure water at a 1:10 volume ratio) was added and the elements trapped by covering with the plasma-activated (O_2, 30 mL/min, 100 W, 30 s, PVA TePla 100) PDMS-replica, followed by a clean (25 x 25) mm cover glass. Finally, the assembly was left on the microscope stage at 30-40°C until excess liquid was fully evaporated.

Confocal laser scanning microscopy

The investigated elements were collected with ultra pure water into rectangular glass capillaries, as described before. To enhance usability, the capillaries were additionally fixed to a microscope slide using clear nail polish (fisher scientific). The confocal laser scanning microscopy images were recorded using a Leica TCS SPE microscope with a 100x magnification objective (HC PL APO CS2 100x/1.4 Oil). Emission wavelength of 503 nm has been applied and the corresponding fluorescence signal has been detected at 508 – 584 nm.

Curvature analysis

The curvature of the conical helices was analyzed using confocal images. Therefore, Z-stack measurements were converted into color depth coded 3D models using the LASX software (Leica Microsystems CMS GmbH). The perspective of observation was rotated to a view along the helix axis (see **Fig. S1i-ii**). The resulting 2D image was exported and loaded in **ImageJ**, where further analysis was done using the Kappa plugin for curvature analysis.[55] Color depth coding in the image was used to identify the wide end of the ribbon. Starting at this point, a B-Spline was drawn, following the edge of the ribbon towards the thin end. The curvature of the B-spline was extracted and plotted against the length, where 0 µm denotes the starting point on the wide end (**Fig S1 iii**). The mean curvature of each conical helix was calculated by averaging all values. A linear fit was applied to the curvature profile, describing the gradient in curvature along the length of the ribbon by the slope *m*.

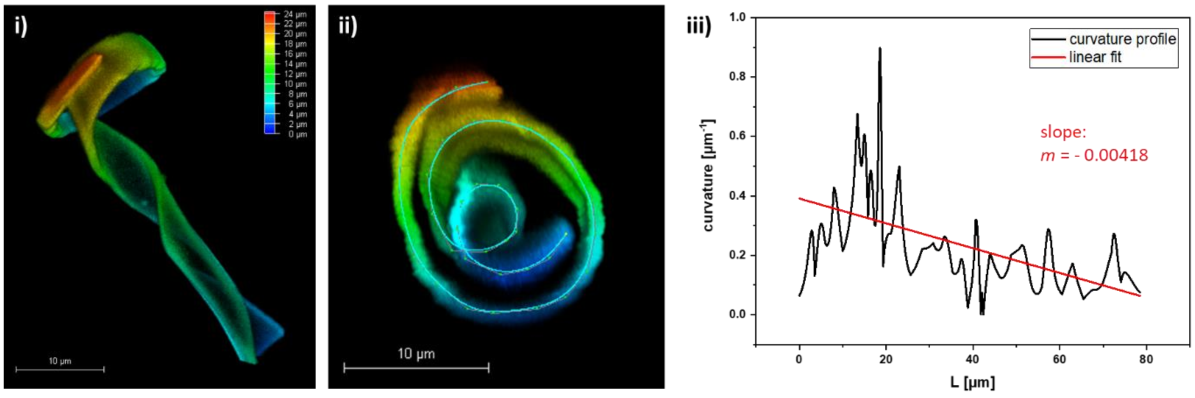

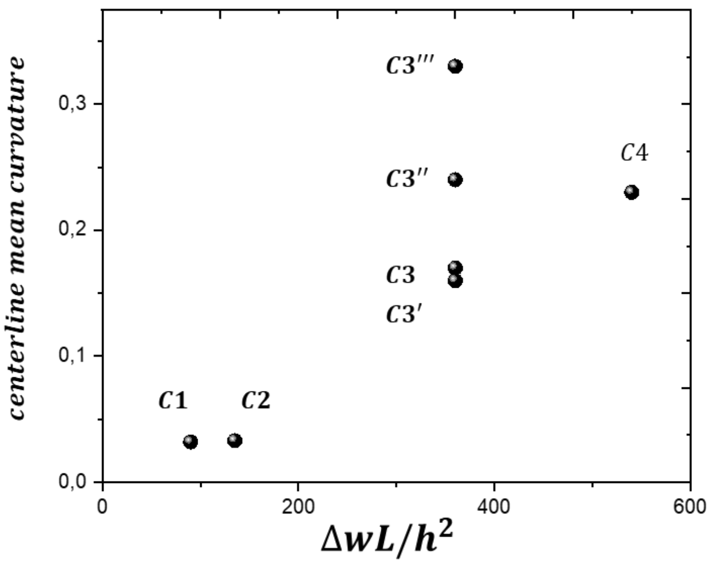

Figure S1: *Curvature analysis of a conical helix. i) A 3D model of a Z-stack measurement from laser scanning microscopy experiment is composed using LAS X software (Leica Microsystems CMS GmbH). ii) The 3D image is rotated to view the object along the axis of the helix. Subsequently, a 2D image is recorded, imported in ImageJ, and a B-Spline is drawn along the edge of the ribbon (blue line). iii) The resulting curvature is plotted against the length of the ribbon from the wide end (w = 5 µm, L = 0 µm) to the thin end (w = 0.5 µm, L = 80 µm). The slope m of the linear fit describes the gradient in curvature of the object.* In order to classify the different conformations we also plotted the mean curvature measured along the length of the ribbon against $\Delta wL/h^2$ Föppl-von-Kármán number which is a geometric parameter how easily a sheet bends.
